# Non-coronary cardiac manifestations of systemic lupus erythematosus in adults: a comparative study

**DOI:** 10.11604/pamj.2019.33.156.18697

**Published:** 2019-07-02

**Authors:** Sameh Sayhi, Nour Gueddich, Rym Dhahri, Najeh Bousetta, Bilel Arfaoui, Nadia Ben Abdelhafidh, Faida Ajili, Bassem Louzir

**Affiliations:** 1 Autoimmune Disease Research Unit (Ur17dn02), Internal Medicine Department, Military Hospital of Tunis, Tunis, Tunisia

**Keywords:** Systemic lupus erythematosus, cardiac manifestations, pericarditis

## Abstract

Cardiac manifestations develop in the majority of patients with systemic lupus erythematosus (SLE) at some time during the course of their disease. This study was designed to assess cardiac abnormalities in patients with SLE by echocardiography and to compare the 2 groups of patients with and without cardiac manifestations. It was a transversal, descriptive study, conducted in the Internal Medicine Department at the Military Hospital of Tunis from January 2016 to June 2018. Eighty lupus patients, diagnosed on the basis of ACR (American college of rheumatology) criteria, were enrolled in the study and were evaluated by standard echocardiography with color Doppler. Out of 80 patients 42 (52%) had abnormal echocardiographic findings. Pericardial effusion was found in 55%, valvular abnormalities in 52% and 38% had pulmonary hypertension. Patients with pleural effusion (45 vs 15%) were more vulnerable to cardiac involvement as well as renal impairment (57 vs 44%). The difference, however, were not statistically significant (p>0.05) in the renal involvement. Active disease with low complement (80%) was associated with higher frequency of cardiac involvement than disease in remission (64%) but the result was not statistically significant (p=0.11). Cardiac abnormalities are very common in lupus patients even when clinically asymptomatic form. Echocardiography is an excellent non-invasive tool for cardiac evaluation. Their research must be systematic with echocardiography in order to reduce subsequent cardiac morbidity and mortality among the lupus patients.

## Introduction

Systemic lupus erythematosus (SLE) is a common chronic multi-system autoimmune disorder of unknown etiology causing injury to many organ systems. It predominantly affects young women. SLE affects 40-200 per 100 000 persons [1], with the higher values seen in black populations. Cardiac manifestations develop in the majority of patients with SLE at some time during the course of their disease [1]. Cardiac involvements in SLE are: valvular disease, most often mitral regurgitation and usually hemodynamically insignificant; pericardial disease, usually an asymptomatic effusion; myocardial dysfunction and coronary artery disease [2]. Current diagnostic methods in particular echocardiography allows early diagnosis of non-coronary cardiac manifestations of systemic lupus erythematosus.

## Methods

The aim of our study was to assess the cardiac abnormalities in SLE patients, the contribution of echocardiography in this disease and to compare the 2 groups of patients with and without non-coronary cardiac manifestations. We have performed a transversal, descriptive study of SLE patients hospitalized in the Internal Medicine Department at the Military Hospital of Tunis between January 2016 and June 2018. Diagnosis of SLE was made according to the criteria of the American college of rheumatology (ACR). All patients underwent a cardiac ultrasound externally or during their stay in our department.

## Results

The patients were 61 females and 19 males (sex-ratio=3) with a mean age of 38 years. We divided the workforce into two groups according to the presence or absence of cardiac manifestations: group 1 (patients with cardiac disease) and group 2 (patients without cardiac disease. The clinical features of the patients of group 1 are summarized in Table 1. Forty-two patients had cardiac involvement (Group 1). They were 33 females and 9 males with a mean age of the disease of 31,8 years (16-80 years) at the beginning of the disease and 41 years at the time of the study. In group 1, 83% were symptomatic. The symptoms were dominated by objectified chest pain (43%). In Doppler echocardiography, pericarditis was found in 23 patients (55%) with a single case of cardiac tamponade. Libman Saks endocarditis and lupus myocarditis were found in one case each. Pulmonary hypertension (HTP) was observed in 16 patients (38%) and valvular disease in 22 patients (52%). Cardiomegaly was observed in 9 patients (21%). Electrical abnormalities were dominated by micro-voltage found in 8 patients. The general symptoms (83%), skin lesions (76%) and musculoskeletal involvement (64%) were the most frequent events associated with the cardiac manifestations in group 1 (Table 1). ANA were positive in 97% of cases and antiphospholipid antibodies in 24%. Prednisone: 1mg/kg/day and immunosuppressive therapy were indicated respectively in 71% and 38% of patients of group 1. The comparative study of the two groups showed that patients with pleural effusion (45 vs 15%) were more vulnerable to cardiac involvement (P=0.004) as well as renal impairment (57 vs 44%) but the difference, however, was not statistically significant here (p>0.05). Rnp antibodies was higher in group 1 and statistically significant (p<0,05). Some antibodies, and antiphospholipid antibodies were higher in group 1 but the difference was not statistically significant here (p>0.05). Active disease with low complement (80%) was associated with higher frequency of cardiac involvement than disease in remission (62%) but the result was not statistically significant (p=0.07) (Table 2).

**Table 1:** Clinical features of patients with cardiac manifestations

Clinical features	Patients (n=42)	n (%)
general signs		15 (36%)
Reached mucocutaneous	Photosensitivity	16 (38%)
	Nasal ulcerations	2 (5%)
	malar rash	13 (31%)
joint damage	arthralgia	19 (45%)
	arthritis	8 (19%)
	Deformation hand Jaccoud	1 (2%)
renal impairment	Glomerular nephropathy	18 (43%)
	Renal Insufficiency	10 (24%)
Serositis	pleurisy	13 (31%)
	ascites	5 (12%)
lung disease	diffuse infiltrative lung disease	5 (12%)
achieved hematologic	neutropenia	5 (12%)
	thrombopenia	5 (12%)
	Heamolytic anemia	6 (14%)
neurological impairment	reached parenchymal	6 (14%)
Events thromboembolic	Thrombosis of the lower limbs	2 (5%)
	pulmonary embolism	2 (5%)
lupus myositis		1 (2,3%)
hepatitis lupus		1 (2,3%)

**Table 2:** Comparison of patients with and without cardiac manifestations

	Group 1 n (42)	Group 2 n (38)	P
Sex (female)	34	32	0.70
General signs	35	29	0.43
Skin involvement	33	29	0.81
Musculoskeletal involvement	27	24	0.91
Achieved hematologic	27	25	0.88
Serositis: pleural effusion	19	6	0.004
Renal impairment	24	17	0.26
Neurological involvement	6	6	0.85
Ac anti DNA natifs	29	26	0.95
Low complement	34	24	0.07
Ac anti Sm	9	4	0.18
Ac anti Rnp	8	1	0.03
Ac antiphospholipid	10	4	0.11
Corticosteroids1mg/kg/j	30	28	0.82
Cyclophosphamide	16	15	0.89

## Discussion

Cardiovascular abnormalities are common in patients with systemic lupus erythematosus [3]. SLE is among systemic diseases most providers of heart disease [4, 5]. It probably develops in nearly all patients suffering from SLE at some time during the course of their disease. All components of the heart can be affected and venous thrombo-embolic complications may cause pulmonary hypertension and right heart failure [6]. Cardiac disease is observed in 30 to 62% by clinical, ultrasound or autopsy diagnostic tool [7, 8]. Its prevalence was 52% in our study, it was 70% in the French study of Badoui *et al* [9] and in 68% in the Polish study of Ostanek *et al* [10]. Pericardial involvement is the most common echocardiographic lesion in systemic lupus erythematosus (SLE) and is the most frequent cause of symptomatic cardiac disease. It is observed in 11 to 54% of the cases [7]. In our study, the frequency of pericarditis was 55% higher than those described in the recent studies. Tamponade occurs in less than 2% and constrictive pericarditis is extremely rare [1, 2]. Cardiac involvement may precede the clinical signs of lupus and it is usually asymptomatic. It is generally diagnosed by echocardiography performed for some other reason, such as suggestive electrocardiographic abnormalities. Pericarditis, as with other types of serositis, most often occurs when SLE is active in other organs as well. In our experience, patients with pleural effusion (45 vs 15%) were more vulnerable to cardiac involvement (P=0.004). Cardiomyopathy is uncommon clinically but autopsy studies found myocardial involvement in 40-50% of patients [1]. Valvular disease is often underdiagnosed because most of the time they are asymptomatic. Pulmonary arterial hypertension (PAH) is a rare complication of systemic lupus erythematosus (SLE) [9]. Its prevalence depends on the method with which it is diagnosed and the tools used for that (echocardiography or heart catheterism). This prevalence varies from 4.2 to 17.5% in the literature when diagnosed by echocardiography and from 0.5 to 9.3% using the right heart catheterism. In our patients, where PAH was defined by a systolic pulmonary arterial hypertension ≥ 30 mmHg in echocardiography, its prevalence was of 38% higher than those of the literature.

## Conclusion

Cardiac abnormalities are very common in lupus patients even when clinically asymptomatic. Echocardiography is an excellent non-invasive tool for cardiac evaluation. Their research must be systematic with echocardiography in order to reduce subsequent cardiac morbidity and mortality among the lupus patients.

### What is known about this topic

- Cardiovascular abnormalities are common in patients with systemic lupus erythematosus;
- Cardiomyopathy is uncommon clinically but autopsy studies found myocardial involvement.

### What this study adds

- This study was designed to assess cardiac abnormalities in patients with SLE by echocardiography and to compare the 2 groups of patients with and without cardiac manifestations;
- Echocardiography is an excellent non-invasive tool for cardiac evaluation even when clinically asymptomatic.

## Competing interests

The authors declare no competing interests.
